# Are neurological and psychiatric disorders different?^†^

**DOI:** 10.1192/bjp.bp.114.158550

**Published:** 2015-11

**Authors:** Anthony S. David, Timothy Nicholson

**Affiliations:** **Anthony S. David**, FRCP, FRCPsych, Timothy Nicholson, MRCP, MRCPsych, Institute of Psychiatry, Psychology and Neuroscience, King's College London, London, UK

## Abstract

There have been recent calls to abandon the distinction between neurological and psychiatric disorders on philosophical and moral grounds. Crossley and colleagues, in this issue, meta-analyse published structural brain imaging data and prove that they are different after all – or do they?

**Figure:**
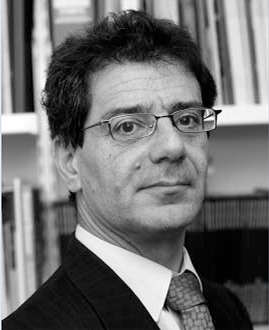


## Background

The study by Crossley and colleagues,^1^ in this issue of the *BJPsych*, is a rare and therefore welcome empirical contribution to the debate, recently revived, on whether the distinction between psychiatric and neurological disorders is real and/or should be abandoned.^2^ Psychiatric disorders are not just ‘mental’ but physical too.

The ‘reality’ of the psychiatry–neurology distinction, refers to the soundness of its theoretical basis. This might be best summarised by the following rule: if a disorder in question is reliably associated with a recognisable pathological process affecting the central nervous system (CNS), then it is neurological. But that of course just begs questions about what is meant by ‘reliably’? Does this mean ‘necessary and sufficient’ to cause the condition? What is ‘recognisable pathology’? Does this refer to the microscopic or macroscopic level? What then do we make of ‘quantitative change’ in the CNS in relation to some normative standard of a given magnitude? Is that ‘pathology’?

If we take schizophrenia, there is ample evidence of quantitative regional change in the CNS thanks to the widespread use of structural magnetic resonance imaging, but only insofar as this is detectable at the group level against a control group and according to some more or less arbitrary statistical threshold.^3^ The same can be said of affective disorder but at a rather lower statistical threshold. So are these neurological disorders? Perhaps, yet we do not see much interest among our neurology colleagues to start seeing these patients or engaging in research on them. What then of Alzheimer's disease? Clearly a neurological disorder and of interest to a few neurologists, but ‘core business’ for the old age psychiatrist and a shared topic of interest for research. Hence, the discussion moves rapidly from philosophy (is the distinction real?), through biology (is there evidence of a pathological process?), to sociology, culture and prejudice (whose job is it? who gets the credit/blame?).

## Crossley *et al'*s meta-analysis

The paper by Crossley and colleagues takes a novel approach.^1^ It is a meta-analysis of studies using voxel-based morphometry (VBM) to measure changes in grey matter in neurological and psychiatric diagnostic groups. So there are studies of diseases as diverse as multiple sclerosis and Huntington's disease, Asperger syndrome, anorexia and Alzheimer's disease, schizophrenia and panic disorder, each comparing a patient group with controls and reporting statistical differences in, strictly speaking grey matter density used as a proxy for volume change, usually with, areas of reduced grey matter mapped onto a brain template. Importantly, these methods look at the whole brain and are not influenced by prior assumptions of where the abnormality might be. The authors applied some basic quality controls over the included studies and applied weightings according to the numbers of participants but were interested in the broad pattern that such large data-sets might reveal.

Now, you might question the whole enterprise. Is it not like going into a general hospital, picking patients from the cardiac ward, the renal unit and the orthopaedic department and sticking them in a scanner? Little surprise then if the results showed that abnormalities tended to centre on (although not being confined to) the heart, the kidney and the skeleton, respectively? But it is not quite the same since both psychiatry and neurology are laying claim to the same organ, the brain. Going along with the idea one might then hypothesise that neurological patients show the bulk of abnormalities in the areas of the brain associated with more ‘basic’ functions: movement, sensation and, with respect to dementia, memory, whereas psychiatry patients show differences in ‘higher’ brain regions associated with self-consciousness and identity (the frontal lobes) or emotion (the ‘limbic system’).

The results were more nuanced. First and foremost, there was a clear and statistically robust difference between the two classes of disorder. Second, the pattern was to some extent in line with predictions but with surprises. Summarising the data both quantitatively and qualitatively, the basal ganglia and insula clearly fell on the neurological side of the divide as did primary sensory and motor networks. However, so did the dorsal prefrontal ‘executive’ region, which might not have been envisaged. On the other hand the area that came out as clearly ‘psychiatric’ was the medial prefrontal region, an area with a growing reputation for functions concerning self-reflection and social cognition.^4^ This region is part of the so-called ‘default mode network’, thought to be involved in awareness and stimulus-independent thought,^5^ which was generally less implicated in studies of neurology patients and includes attentional systems reaching back to the posterior cingulate cortex. An unexpected and frankly odd finding was that regions of the visual association cortex were significantly more allied with psychiatric disorders. These included the lingual gyrus, which may after all be genuinely important given its role in face perception. The temporal cortex was, perhaps predictably, the truly contested area – containing both the hippocampus (reflecting the arbitrariness of regarding dementia as neurological or psychiatric) and components of the limbic system, as well as being a key area of epilepsy pathology, such as mesial temporal sclerosis.

## Context

This is not the first global look at grey matter across diagnoses. A recent meta-analysis of VBM studies with similar methods has just been published by Goodkind and colleagues, this time focusing just on psychiatric (Axis I) diagnoses, but including all published data rather than just representative samples.^6^ This study found reduced grey matter in both anterior cingulate and insula cortices to be common across all disorders, concluding these areas could represent a ‘shared neural substrate’ for mental illness. It is interesting to note that the insula in this study was also found to be a key psychiatric region, whereas it came out as associated with neurological disease in Crossley *et al*'s study. Again, this ambiguity is perhaps not surprising given the large body of evidence linking the insula with emotion processing, motor function and – a true interface between mind and body – introception.^7^

There are a few limitations to get out of the way. The studies do not take into account medication. There is controversial evidence that antipsychotic medication can alter brain structure, for example increasing volume loss.^8^ Somewhat less controversially, traditional antipsychotics have been found to cause increases in basal ganglia volumes.^9^ Hence, what we may be seeing here is the interaction between diagnosis and medication. Also, neither study was confined to grey matter and as psychiatric neuroimaging techniques advance and spread (for example encompassing more diffusion tensor imaging) we would expect more studies on white matter and connectivity to be published and to contribute to this debate.^10^

## Conclusions

The results may be taken as a slap in the face to the distinction-abolitionists, yet such individuals might take heart in them in that there is no implicit hierarchy in what emerges as the brain-based hallmark of neurological versus psychiatric conditions; they both involve the functionally interesting parts of the brain, it is just that they are, quite subtly, different.

Perhaps one lesson that we can all take from this novel and ingenious work is that we need to continue to use every ounce of our brains to answer the question of what makes a psychiatric disorder. As McHugh & Slavney^11^ wrote in their thoughtful *The Perspectives of Psychiatry*:

‘In the everyday world of the clinic, psychiatrists are distinguished from other medical specialists not because they are concerned with “minds” rather than “bodies”, but because they focus on complaints appearing in people's thoughts, perceptions, moods, and behaviours rather than their skins, bones, muscles and viscera … The diagnostic process may be difficult, but causal explanations are always complex and depend on the physician's capacity to evaluate issues ranging from intermediary metabolism (a “body” issue) to interpersonal misunderstanding (a “mind” issue). Psychiatric concerns thus extend from the ultrastructure of the body to the relationship of groups of minds within a social context.’
